# Cherry-picking of evidence fails to accurately show extent of overdiagnosis: Mammographic screening harms understated

**DOI:** 10.1002/jmrs.13

**Published:** 2013-05-14

**Authors:** Emma Woolley

**Affiliations:** Radiographer & Mammographer3/11, Cooling Place Florey, Act 2615, Australia

Re: Lee W, Peters G. Mammographic screening for breast cancer: A review. *J Med Radiat Sci* 2013; **60**(1): 35–9.

It was with disappointment that I read the review of mammographic screening by Lee and Peters in Volume 60, Issue 1 of the *Journal of Medical Radiation Sciences*. The article clearly demonstrated bias which is inherent when staff of a program reviews the efficacy of their own program, resulting in findings incongruent with those found by those without vested interests.

Lee and Peters reported that 2–2.5 deaths from breast cancer are prevented for each case of overdiagnosis. This was the finding of the EUROSCREEN Working Group, a group comprised of several prominent figures in the world of mammography, including the eminent Prof. Lázsló Tabár.^1^ This study was widely criticized at the time of its publication in 2010, for using inappropriate methods, including extrapolations of the data far beyond what could suitably be inferred.

The findings of the EUROSCREEN Working Group contrast sharply with a 2012 independent review into the NHS Breast Screening Program in the United Kingdom, commissioned by Cancer Research UK and the Department of Health (England), and found that for every 1000 women screened, five deaths from breast cancer are prevented and 17 women are overdiagnosed.^2^

The findings quoted by Lee and Peters are similarly not congruent with the findings of the Nordic Cochrane Centre in Denmark,^3^ which has persistently completed Cochrane reviews of the literature around breast screening, and concluded that the chance that a woman will benefit from attending screening is very small, and considerably smaller than the risk that she may experience harm.

Overdiagnosis is not a simple matter of informed consent, as described by the authors. Overdiagnosis is harm being caused to women by direct involvement in the BreastScreen Australia program – otherwise healthy women will have either a part of their breast or the whole breast removed, and they will often receive radiotherapy and sometimes chemotherapy. That we cannot tell who is being overdiagnosed is not an excuse to inflict this harm.

The authors failed to state their bias in the article. Experts can become a source of bias simply because they are experts: employees of BreastScreen Australia, much like Tabár and his EUROSCREEN Working Group, are unlikely to ever publish findings that doubt the efficacy of their own programs.

It takes great bravery and humility to accept that a program which has received as much financial and political investment as BreastScreen Australia might be doing more harm than good. Had Lee and Peters reviewed all of the current evidence on overdiagnosis, the picture painted by the review would have been quite different.
